# Assessing equity of healthcare utilization in rural China: results from nationally representative surveys from 1993 to 2008

**DOI:** 10.1186/1475-9276-12-34

**Published:** 2013-05-20

**Authors:** Zhongliang Zhou, Yanfang Su, Jianmin Gao, Benjamin Campbell, Zhengwei Zhu, Ling Xu, Yaoguang Zhang

**Affiliations:** 1 School of Public Policy and Administration, Xi’an Jiaotong University, 28 Xianning West Road, Xi’an 710049, China; 2 Department of Global Health and Population, Harvard School of Public Health, 677 Huntington Avenue, Boston, MA 02115, USA; 3 Bryn Mawr College, 101 N Merion Avenue, Bryn Mawr, PA 19010, USA; 4 Center of Health Statistics and Information, Ministry of Health, 1 Xizhimen Nanlu, Beijing 100044, China

**Keywords:** Inequity, Realized healthcare utilization, Need-predicted healthcare utilization, Need-standardized healthcare utilization, Concentration index

## Abstract

**Background:**

The phenomenon of inequitable healthcare utilization in rural China interests policymakers and researchers; however, the inequity has not been actually measured to present the magnitude and trend using nationally representative data.

**Methods:**

Based on the National Health Service Survey (NHSS) in 1993, 1998, 2003, and 2008, the Probit model with the probability of outpatient visit and the probability of inpatient visit as the dependent variables is applied to estimate need-predicted healthcare utilization. Furthermore, need-standardized healthcare utilization is assessed through indirect standardization method. Concentration index is measured to reflect income-related inequity of healthcare utilization.

**Results:**

The concentration index of need-standardized outpatient utilization is 0.0486[95% confidence interval (0.0399, 0.0574)], 0.0310[95% confidence interval (0.0229, 0.0390)], 0.0167[95% confidence interval (0.0069, 0.0264)] and −0.0108[95% confidence interval (−0.0213, -0.0004)] in 1993, 1998, 2003 and 2008, respectively. For inpatient service, the concentration index is 0.0529[95% confidence interval (0.0349, 0.0709)], 0.1543[95% confidence interval (0.1356, 0.1730)], 0.2325[95% confidence interval (0.2132, 0.2518)] and 0.1313[95% confidence interval (0.1174, 0.1451)] in 1993, 1998, 2003 and 2008, respectively.

**Conclusions:**

Utilization of both outpatient and inpatient services was pro-rich in rural China with the exception of outpatient service in 2008. With the same needs for healthcare, rich rural residents utilized more healthcare service than poor rural residents. Compared to utilization of outpatient service, utilization of inpatient service was more inequitable. Inequity of utilization of outpatient service reduced gradually from 1993 to 2008; meanwhile, inequity of inpatient service utilization increased dramatically from 1993 to 2003 and decreased significantly from 2003 to 2008. Recent attempts in China to increase coverage of insurance and primary healthcare could be a contributing factor to counteract the inequity of outpatient utilization, but better benefit packages and delivery strategies still need to be tested and scaled up to reduce future inequity in inpatient utilization in rural China.

## Introduction

The United Nations proposed the Millennium Development Goals (MDGs), which aim to eradicate extreme poverty and improve health conditions by 2015. In addition, the World Bank is committed to improving the health of the poor and ending extreme poverty by 2030 [1]. These initiatives reflect a global effort to reduce socioeconomic and health inequity and to help the least well off. Efforts have been made to measure health equity in developing countries, and one important metric of equity is utilization of healthcare. For example, between 1998 and 2008, Brazil became increasingly equitable in the utilization of healthcare services, representing a shift from largely pro-rich utilization to only a slightly pro-rich utilization of health services [2]. Furthermore, Prinja et al. found that in three northern states in India, trends in healthcare utilization show an increasingly pro-rich distribution and low rates of utilization by the poor [3]. However, they also showed that public sector hospitalizations were mostly pro-poor, suggesting that the public sector could be a venue for reducing inequity in these regions. Lastly, in Malawi, Zere et al. found that healthcare utilization was pro-rich and that it was widening during the period 1992–2004 [4]. As these studies show, measuring trends in equity of healthcare utilization can illuminate whether a country is meeting the health needs of the poorest.

China is another country that historically has major disparities but is undergoing major health and economic transitions [5]. In rural China, the gap between the rich and the poor has been expanded, even though the rural residents’ overall economic situation improved quickly in the last 30 years. And the disparity of health service utilization increased among the rural residents with different economic levels. The fourth National Health Service Survey shows that the number of patients in the poorest group who should be hospitalized but have not is 2.1 times as large as the patients in the richest group [6]. China has undergone transformational health reform since 2009. At the heart of this reform were the establishment of a national primary healthcare system and the enhancement of insurance programs targeting low-income citizens [7]. Nearly 100% of the country is covered up to date. Reform has increased insurance coverage for the poor, but questions remain about how equity in health service delivery and utilization has changed over time.

In resource allocation, healthcare is delivered distinctively according to difference in need, purchasing power, age, gender, amongst other factors. The inequality of healthcare utilization does not necessarily lead to inequity. The inequality of healthcare utilization refers to the disparities in utilization of health services, which may be governed by healthcare need, or by other factors. For an inequality to be interpretable as an inequity, differential need must be taken to into account [8,9]. The interpretation of equity in healthcare utilization is that health care ought to be allocated on the basis of health need, rather than on the basis of other characteristics such as purchasing power, age or gender [10]. Therefore, if healthcare utilization is negatively associated with need for healthcare, the inequality in healthcare utilization is considered as inequity [11], which is an ethical judgment. People with the same needs of healthcare ought to have the same chance to access healthcare, irrespective of their socioeconomic status, which is defined as “horizontal equity” [12]. Alternatively, “vertical equity” measures the dependence of the quantity of healthcare service on the quantity of need for healthcare [13]. Specifically, individuals with higher need for healthcare ought to have more access to healthcare. In empirical studies, technical skills to measure horizontal inequity of healthcare utilization has been advanced [12,14]; however, measurement of vertical inequity of health utilization remains underdeveloped [15]. Our study applies the well-developed measures to empirically estimate horizontal inequity of healthcare utilization in rural China.

The method to measure horizontal inequity of healthcare utilization was first proposed by Le Grand in 1978 [16]. In his analysis of the equity of health care delivery, he performed two types of calculation. In the first, he computed the cost per person reporting illness in each socio-economic group (SEG), and in the second calculation he computed the share of expenditure received by each SEG and compared this with the group’s share of ill-health. However, Le Grand’s approach to measure the horizontal inequity of healthcare utilization was criticized by some researchers because of its deficiencies that it focuses exclusively on the extreme classes and fails to take into account their relative sizes and the confounding effects of demographic factors [12,17,18]. Based on Le Grand’s approach, Wagstaff et al. suggested the concentration curve approach in 1989 [19], which overcomes some limitations of Le Grand’s range measures but does nothing to tackle the other deficiencies, such as the confounding effects of demographic factors. Two years later, the direct standardization method was suggested by Wagstaff et al. [20], which is to divide the sample into income groups and then compute need-standardized medical care figures for each income group. However, this method has a major disadvantage in that it requires the use of grouped data and its usefulness is therefore limited by the fact that the value of concentration index depends on the number of income groups. In order to remedy the disadvantage, Wagstaff et al. developed two advanced methods, the indirect standardization method [20] and the method of decomposing concentration index [21], to measure the horizontal inequity of healthcare utilization. These two methods are used extensively by other researchers.

In China, Yu et al. [22] first analyzed disparity of annual hospitalization rate and the average expenses of hospitalization in 1997, which demonstrated inequality rather than the inequity of healthcare utilization. For more than a decade, most research mainly explored the inequality of healthcare utilization [22-26]. A turning point occurred when Zhao et al. [27] applied the method of indirect standardization to the study of horizontal inequity of healthcare utilization, including outpatient and inpatient services, for the residents in Gansu Province, China. Since then, more studies investigated horizontal inequity of healthcare utilization among rural residents in China [28-31]. However, it was limited to a particular place and cross-sectional time; thus, the results could not represent the whole nation of China. Nor did they show the trend of inequity over time. The aim of this study is to estimate inequity of healthcare utilization using nationally representative samples from 1993 to 2008 in rural China. The indirect standardization method developed by Wagstaff et al. will be employed to measure the horizontal inequity of healthcare utilization in this study [10].

### Data

In order to assess inequity and further plot the trend of inequity over time, we obtained access to the nationally representative household survey, National Health Services Surveys (NHSS), which has been collected by the Center for Health Statistics and Information, Ministry of Health, in China, from 1993 to 2008.

### Sampling

Through a four-stage stratified sampling procedure, samples have been randomly selected for household surveys in NHSS and we focus on the rural component of the survey. In the first stage, 65 rural counties were randomly selected. In the second stage, 5 townships in each county were randomly chosen. In the third stage, 2 villages in each township were randomly selected. Finally, around 60 households were chosen in each village. The counties, towns, and villages selected in four NHSSs remained the same; however, households were different in each survey. Therefore, NHSS is panel data at village or upper levels but only repeated cross-sectional data at an individual level. Xu and Gao elaborated the sampling strategy and the process of quality control of NHSS [32,33].

Table 1 shows the total numbers of rural households sampled from 1993 to 2008, which were 38,775 in 1993, 40,209 in 1998, 40,212 in 2003, and 39,054 in 2008. Only people who were 15 years old and older were included in this study and this included 113,458 in 1993, 119,037 in 1998, 112,054 in 2003, and 103,773 in 2008. The average household size, calculated through dividing the number of individuals by the number of households, is 4.15, 4.01, 3.58, and 3.31 from 1993 to 2008. It indicated that household size shrink over time. Therefore, in measuring income, we use per capita consumption expenditure rather than household consumption expenditure to rule out variation in household size. Information in NHSS includes demographic characteristics, self-reported health status, illness, and chronic disease, consumption expenditure, healthcare utilization, and health insurance scheme, etc. Cross-sectional data from the year of 1993, 1998, 2003, and 2008 are analyzed through separated regressions.

**Table 1:** Sample size

	**1993**	**1998**	**2003**	**2008**
Households	38,775	40,209	40,212	39,054
Individuals (≥15 yrs)	113,458	119,037	112,054	103,773

Resource: National Health Services Survey, from 1993 to 2008.

### Variables

In quantifying healthcare inequity, we utilize three groups of variables: healthcare utilization variables, healthcare need variables and control variables [30] (Table 2).

**Table 2:** Variables

**Category**	**Variables**
Healthcare utilization	The probability of outpatient visit, The probability of inpatient visit
Healthcare need	Sex, Age, Illness in last two weeks, Chronic disease, Sick days, Confined-to-bed days, off-work Days
Control variables	Smoking behavior, Drinking behavior, Marital status, Education level, Employment, Region, Health insurance, Price of outpatient visit, Price of inpatient visit, and Income

Healthcare utilization is measured in two perspectives: the probability of outpatient visit and the probability of inpatient visit. The probability of outpatient visit refers to the probability of using outpatient service in the last two weeks, which is based on the question in questionnaire “did you visit a doctor in the past two weeks”. The probability of outpatient visit was in the range from 7.93% to 9.13% from 1993 to 2008. The probability of inpatient visit means the probability of using inpatient service in the previous year, which is based on the question “have you been hospitalized in the past year”. The probability of inpatient visit increased from 3.37% to 6.11% from 2003 to 2008 (Table 3).

**Table 3:** Description of variables in the year of 1993, 1998, 2003 and 2008 (Percentage/means)

**Variables**	**Description**	**1993**	**1998**	**2003**	**2008**
Healthcare utilization					
The probability of outpatient visit	The probability of utilizing outpatient service in past two weeks	8.09	9.13	7.93	8.46
The probability of inpatient visit	The probability of utilizing inpatient service in the past year	3.33	3.07	3.37	6.16
Healthcare need					
Sex	Male	50.1	50.9	50.4	49.7
	Female*	49.9	49.2	49.6	50.3
Agea	Age 15-34*	49.9	46.6	37.5	31.0
	Age 35-44	20.2	19.4	21.5	22.7
	Age 45-54	12.3	15.1	19.7	19.2
	Age 55-64	9.5	9.4	10.9	15.0
	Age 65+	8.2	9.5	10.4	12.2
Illness	Ill in last two weeks	12.7	13.8	14.9	18.4
	Not ill in last two weeks*	87.3	86.2	85.1	81.6
Chronic disease	Chronic disease	14.5	13.5	13.2	17.3
	No chronic disease*	85.5	86.5	86.8	82.7
Sick days	Sick days in last two weeks	8.5	8.7	7.9	8.5
Confined-to-bed days	Days staying in bed because of illness in last two weeks	1.1	1.0	1.3	1.2
Off-work days	Days off work or off school in last two weeks because of illness	2.3	2.5	1.8	1.4
Control variables					
Smoking	Smoke	32.5	29.5	26.8	26.0
	Never smoke*	67.5	70.5	73.2	74.0
Drinking	Drink alcohol	18.9	16.2	15.7	13.1
	Never drink alcohol*	81.1	83.8	84.3	86.9
Marital status	Unmarried*	22.9	20.7	18.4	16.5
	Married	70.6	72.6	74.8	75.3
	Divorced	0.5	0.7	0.7	1.2
	Widowed	6.0	6.0	6.1	7.0
Education	Illiterate*	30.2	24.9	22.8	19.0
	Elementary school	33.8	33.1	31.2	31.5
	Secondary school	28.9	33.7	36.1	37.8
	High school	6.8	7.7	8.9	10.2
	University	0.4	0.6	1.1	1.5
Employment	Unemployment*	0.4	7.0	4.0	12.9
	Farmer	72.3	75.4	72.3	64.4
	Student	4.7	5.4	6.3	7.2
	Other occupations	22.7	12.2	17.5	15.5
Region	Eastern*	31.6	31.0	31.2	31.0
	Central	28.4	26.7	26.8	27.4
	Western	40.0	42.3	42.0	41.6
Health insurance	Uninsured*	82.7	87.5	87.5	6.5
	CMS/NCMS	10.5	7.1	9.7	90.2
	Other health insurance	6.8	5.4	2.8	3.3
Outpatient price	Median price of outpatient service (total expenses). Natural log of outpatient price is introduced in regression models.	10	21	39	72
Inpatient price	Median price of inpatient service (total expenses). Natural log of inpatient price is introduced in regression models.	345	980	1473	1977
Income	Consumption expenditure per capita. Natural log of income is introduced in regression models.	1335	1594	1786	3056

Note: * Reference groups. Per capita consumption expenditures in the year of 1998, 2003 and 2008 are deflated to the year of 1993 using consumer price index (CPI) at county level.

Healthcare need is an elusive concept that has been interpreted in a variety of ways. According to Culyer and Wagstaff, a good definition should include consideration of key variables such as sex, age and wellness of the residents [34]. As Table 3 shows, the age structure of Chinese rural residents had changed dramatically from 1993 to 2008. Specifically, China has an aging population and healthcare needs are evolving to respond to this demographic trend. Regarding illness in the last two weeks, the percentage of ill residents in the last two weeks had increased from 12.7% in 1993 to 18.4% in 2008. The percentage of the residents who had suffered from chronic diseases had little change from 1993 to 2003; however, it increased from 13.2% to 17.3%, from 2003 to 2008. Sick days and confined-to-bed days had been almost the same from 1993 to 2008. However, rural residents in China experienced less off-work days due to illness in 2008 compared to the year of 1993, 1998, and 2003.

Control variables in quantifying healthcare inequity include: smoking behavior, drinking behavior, marital status, education level, employment, region, health insurance, price of outpatient visit, price of inpatient visit, and income. The statistics of these variables are summarized in Table 3. For the health insurance, rural residents enrolled in health insurance were covered by the cooperative medical system (CMS) in 1993, 1998 and 2003 and by the new rural cooperative medical system (NCMS) in 2008. No more than 10.5% of the rural population was covered by CMS; the coverage rate of NCMS was over 90% in 2008 (Table 3). The price of outpatient and inpatient visits was measured by the median of outpatient and inpatient expenses per visit in the county, and it is implicitly assumed that patients within the same county face the same healthcare price. Income was measured by self-reported consumption expenditure [35]. The per-capita consumption expenditure of rural residents had increased from RMB1335 (214USD) in 1993 to RMB3056 (490USD) in 2008, which nearly increased by 230%, meanwhile, the price of outpatient visit and that of inpatient visit increased by 620% and 473%, respectively (Table 3).

## Methods

Three groups of healthcare utilization were distinguished in quantitative analysis: realized healthcare utilization, need-predicted healthcare utilization, and need-standardized healthcare utilization. Realized healthcare utilization refers to actual healthcare utilization, which was collected in household survey. Need-predicted healthcare utilization is used to capture variation in healthcare utilization predicted only by needs for healthcare, which is calculated through statistical modeling. Need-standardized healthcare utilization is used to capture the gap between realized healthcare utilization and need-predicted healthcare utilization.

The income-related inequity of healthcare utilization is measured in two steps. First, based on the realized healthcare utilization in NHSSs, the Probit Regression Model is applied to generate need-expected healthcare utilization, which is an essential part to calculating need-standardized healthcare utilization through the method of indirect standardization. Second, concentration index is measured for need-standardized healthcare utilization. The concentration index of need-standardized healthcare utilization is to reflect the income-related horizontal inequity of healthcare utilization.

### Standardization of health utilization

As the utilization of healthcare is a binary response, Probit regression model is used with the probability of outpatient visit and the probability of inpatient visit as the dependent variables to indirectly standardize the healthcare service utilization [36]. As the standardization of health utilization holds for a linear model of healthcare, the linear approximation to the Probit model is made by estimating the partial effects evaluated at the means [8].

Probit regression is specified as:

(1) $$y_{i}=G\;\left(\alpha +\sum {}_{j}\beta_{j}x_{\mathrm{ji}}+\sum {}_{k}\gamma_{k}z_{\mathrm{ki}}\right)\;\epsilon_{i}$$

G is a functional transformation, y is the dependent variable, x_ji_ are needs variables, and z_ki_ are control variables.

First of all, need-based healthcare utilization is predicted. Need-predicted healthcare utilization is only impacted by the variation of needs of healthcare, keeping the control variables constant at the level of mean.

(2) $${\hat{y}}_{i}^{x}=G\;\left(\hat{\alpha }+\sum {}_{j}{\hat{\beta }}_{j}x_{\mathrm{ji}}+\sum {}_{k}{\hat{y}}_{k}{\bar{z}}_{k}\right)$$

The disparity in the gap between the need-predicted healthcare utilization and realized healthcare utilization is the preliminary measure of inequity of healthcare utilization. Technically, in the method of indirect standardization, standardized healthcare utilization is calculated by adding the mean of predicted healthcare utilization:

(3) $${\hat{y}}_{i}^{\mathrm{IS}}=y_{i}-{\hat{y}}_{i}^{x}+\frac{1}{n}\sum_{i=1}^{n}G\;\left(\hat{\alpha }+\sum {}_{j}{\hat{\beta }}_{j}x_{\mathrm{ji}}+\sum {}_{k}{\hat{y}}_{k}{\bar{z}}_{k}\right)$$

where ${\hat{y}}_{i}^{\mathrm{IS}}$ is standardized healthcare utilization. *n* is sample size.

From the societal perspective, the total standardized healthcare utilization equals the total realized healthcare utilization, which means inequity could be improved without demanding more healthcare resource.

(4) $$n{\hat{y}}_{i}^{\mathrm{IS}}=ny_{i}-n{\hat{y}}_{i}^{x}+\sum_{i=1}^{n}G\;\left(\hat{\alpha }+\sum {}_{j}{\hat{\beta }}_{j}x_{\mathrm{ji}}+\sum {}_{k}{\hat{y}}_{k}{\bar{z}}_{k}\right)$$

In other words, re-distribution of existing healthcare resources towards unmet need of healthcare improves equity. The more healthcare allocated to the needed, the less inequity of healthcare utilization.

### Estimate of concentration index

Concentration index is employed to measure the degree of income-related inequity of healthcare utilization. The concentration index is calculated through:

(5) $$C=\frac{2}{\mu }\mathrm{cov}\left(h,r\right)$$

where *C* is concentration index, *h* is need-standardized healthcare utilization,*μ* is the mean of need-standardized healthcare utilization, *r* is the fractional rank of income, ranging from 0 to 1. The rank of the *i* individual is: *r*_*i*_ = *i/N*, in which *N* is the number of individuals.

The standard deviation of concentration index is estimated through:

(6) $$\mathrm{var}\;\left(\hat{C}\right)=\frac{1}{n}\left[\frac{1}{n}\sum_{i=1}^{n}a_{i}^{2}-{\left(1+C\right)}^{2}\right]$$

in which $a_{i}=\frac{h_{i}}{\mu }\left(2r_{i}-1-C\right)+2-q_{i-1}-q_{i}$ and $q_{i}=\frac{1}{\mathrm{\mu n}}\sum_{j=1}^{i}h_{j}$.

Concentration index ranges from −1 to +1 [17]. Positive concentration index signals pro-rich inequality, which means the high-income people utilize more healthcare than the low-income counterpart. Meanwhile, the concentration index is negative if the low-income group utilizes more healthcare than the rich counterpart [37]. In extreme cases, the concentration index reaches −1 if all healthcare resources are utilized by the poor; whereas, the index is +1 if the rich are favored in healthcare utilization. Healthcare is equitably utilized by the poor and the rich when the index is 0. Both the point estimate and the confidence interval of the concentration index are measured using nationally representative data.

## Results

Table 4 shows concentration index of need-standardized healthcare utilization, for outpatient service and inpatient service, respectively. All concentration indices are significantly different from zero at 95% confidence level. All point estimates of concentration indices are positive, except that of need-standardized utilization of outpatient service in 2003. The concentration index of need-standardized outpatient utilization is 0.0486[95% confidence interval (0.0399, 0.0574)], 0.0310[95% confidence interval (0.0229, 0.0390)], 0.0167[95% confidence interval (0.0069, 0.0264)] and −0.0108 [95% confidence interval (−0.0213, -0.0004)] in 1993, 1998, 2003 and 2008, respectively. The concentration index of need-standardized inpatient utilization is 0.0529 [95% confidence interval (0.0349, 0.0709)], 0.1543[95% confidence interval (0.1356, 0.1730)], 0.2325 [95% confidence interval (0.2132, 0.2518)] and 0.1313[95% confidence interval (0.1174, 0.1451)] in 1993, 1998, 2003 and 2008, respectively.

**Table 4:** Concentration index of standardized healthcare utilization

	**Utilization of outpatient service**	**Utilization of inpatient service**	**P**
1993	0.0486*	0.0529*	>0.001
	(0.0045)	(0.0092)	
1998	0.0310*	0.1543*	<0.001
	(0.0041)	(0.0095)	
2003	0.0167*	0.2325*	<0.001
	(0.0050)	(0.0099)	
2008	−0.0108*	0.1313*	<0.001
	(0.0053)	(0.0070)	
P	<0.001	<0.001	

Note: Standard errors of concentration index are in the parentheses. * Significant at 5%. *T*-test is used to compare the statistical difference of concentration indexes between the utilization of outpatient service and utilization of inpatient service within each year. Analysis of variance is used the compare the statistical difference of concentration indexes among the year of 1993, 1998, 2003 and 2008.

**Figure 1:**
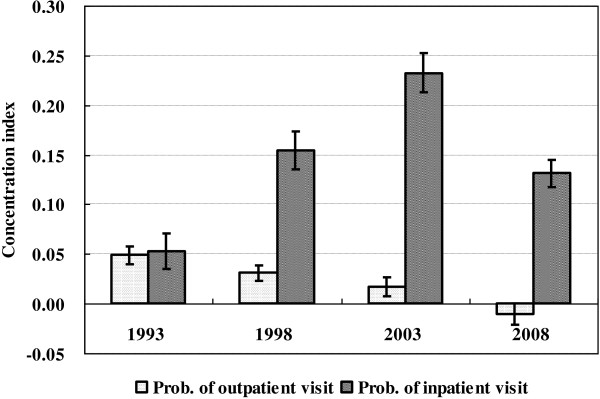
Concentration index of standardized outpatient and inpatient utilization from 1993 to 2008.

Figure 1 visualizes the magnitude and trend of concentration indices for utilization of outpatient and inpatient services over fifteen years. In 1993, concentration index for utilization of outpatient service is slightly lower than that for utilization of inpatient service; however, the difference is statistically insignificant. In the year of 1998, 2003, and 2008, concentration indices for outpatient service are significantly lower than that for inpatient service.

For outpatient services, the point estimates of concentration index became smaller and smaller but remained positive from 1993 to 2003, which changed the sign into negative in 2008. In terms of confidence intervals, concentration index in 1993 was significantly different from that in 1998. Confidence intervals of concentration indices in 1998 and 2003 were overlapped, which indicates a statistically insignificant difference between two years. Concentration index in 2003 was significantly different from that in 2008.

For inpatient services, the point estimates of concentration index remained positive and became greater and greater from 1993 to 2003; however, the value of point estimate dropped in 2008. In terms of confidence intervals, concentration indices in the year of 1993, 1998, and 2003 were significantly different from each other. Nonetheless, the confidence interval of concentration index in 1998 was overlapped with that in 2008, which indicates a statistically insignificant difference between two years.

## Discussion

In this section, we first discuss the boundaries of this study and we further the discussion by pointing out the limitations.

First, in calculating concentration index, difference in income is presented in ordinal perspective. Although rural residents’ income in 2008 was tripled compared to the year of 1993 [38], the variation of income in absolute terms cannot be captured by concentration index. The Gini coefficient, the measure of income inequity, increased continuously from 1993 to 2008 [38]; however, how income was distributed in the population cannot be captured by concentration index, either. The assessment of income-related inequity of healthcare utilization is a static analysis. Impoverishment due to medical expenses was not addressed in assessing concentration index [39,40], let alone the dynamic feedback of medical impoverishment on ordinal income.

Second, the variables of interests, i.e., utilization of outpatient service and utilization of inpatient service, are binary. In estimating inequity, the lower and upper bounds of the concentration index are μ − 1 and 1 − μ, respectively, where μ is the mean of the variable of interest [41]. It is a concern that the mean of the variable empirically restricts the range of the possible values of the concentration index. Specifically, when the mean is zero, the concentration index ranges from −1 to 1. When the mean is 0.5, the concentration index ranges from −0.5 to 0.5. Obviously, the range shrinks when the mean increases. The concentration index is restricted to be zero when the mean of the variable is one. In this study, the means of the variables of interest are relatively small and the ranges of possible values for the concentration index are wide (Table 5).

**Table 5:** The means and the ranges of concentration index for outpatient and inpatient utilization from 1993 to 2008 (%)

	**1993**	**1998**	**2003**	**2008**
The probability of outpatient visit	8.09	9.13	7.93	8.46
	(−0.919, 0.919)	(−0.909, 0.909)	(−0.921, 0.921)	(−0.915, 0.915)
The probability of inpatient visit	3.33	3.07	3.37	6.16
	(−0.967, 0.967)	(−0.969, 0.969)	(−0.966, 0.966)	(−0.938, 0.938)

Note: Ranges of concentration index are in the parentheses.

Third, for both outpatient visits and inpatient visits, the medians of medical cost per visit in each county were taken as the proxies of the perceived price of healthcare. Intuitively, given that patients are price takers, demand for healthcare is affected by the median of medical cost as the perceived price; in turn, individual demand for healthcare has almost no impact on the median of medical cost per visit at county level given large sample sizes in each county. Furthermore, even though price is constructed at the county level to enhance exogeneity, it is still possible to have other sources of confounders from both demand and supply sides. For example, from the supply side, the adaptation of new technology biases up the impact of price on demand for healthcare. However, from the demand side, lowered copay biases down the impact of price on demand for healthcare. The alternative is to take medical costs from individuals as the proxies, which is problematic. Utilization of healthcare by each individual impacts his or her medical cost. In other words, individual medical cost is endogenous to utilization of healthcare; therefore, individual medical cost is not a feasible independent variable. Overall, it is the most feasible to have the medians of medical cost per visit as the proxies to empirically estimate price of healthcare in rural China.

Fourth, this study is restricted to a population of 15 years old or older; therefore, we had no information with regard to equity of healthcare utilization for infants or children under 15.

The conclusiveness of results in the last section is restricted by two main limitations in our analysis.

First, needs-related variables, such as illness in the last two weeks and chronic disease, might suffer from self-reporting bias. Specifically, defining acute illness and chronic diseases depends on access to health information, health consciousness, and healthcare utilization. The rich are more likely to report acute illness and chronic disease due to better access to health information and with a higher chance to utilize healthcare. For example, given that two individuals both acquire tuberculosis and experience coughing, the rich individual might be diagnosed by visiting a doctor and define himself ill. However, the poor individual might be tolerant to coughing, without reporting any illness. If the poor rural residents remain untested for blood pressure, blood sugar, and lipids, the status of chronic disease cannot be accurately reported. Therefore, needs of healthcare for the poor might be underestimated. Consequently, the pro-rich inequity of healthcare service utilization may be underestimated.

Second, the data from NHSS are repeated in a cross-sectional fashion. The trend of inequity might be impacted by variation in sampling. The magnitudes of inequity between outpatient and inpatient services in a specific year are more comparable than the trend of inequity of healthcare utilization over time. For the magnitude of inequity and its trend, analyses on causes are beyond the capacity of the data. Due to lack of identification of causes, policy suggestions also are not discussed here. While it is possible to identify the causes of inequity with panel data, it is still rigorous to restrict the analysis on the magnitude and trend of inequity for repeated cross-sectional data. Policy implications are a good area for future inquiry by setting the research unit at village or upper levels to create panel data.

## Conclusions

There are several key findings in this study.

First, the concentration index of need-standardized healthcare utilization demonstrates that, except utilization of outpatient service in 2008, utilization of outpatient and inpatient services from 1993 to 2008 were pro-rich, which is consistent with Gao’s study conducted in a single county, Zhen’an [26]. Rich rural residents utilize more healthcare than poor counterparts, given the same needs for healthcare. The exception is that poor rural residents utilized more outpatient service than the rich in 2008.

Second, in terms of the trend, inequity of outpatient utilization in rural China gradually reduced from 1993 to 2008. Utilization of outpatient service became more equitable and started to be pro-poor in 2008. The inequity of inpatient utilization dramatically increased from 1993 to 2003 and decreased afterward. The magnitude of inequity remained large over fifteen years.

Third, inequity in utilization of outpatient service became distinctive from inequity in utilization of inpatient service over fifteen years. Starting at almost the same level of inequity in 1993, inequity of inpatient utilization had sharpened and inequity of outpatient utilization had decreased in the following ten years. Even though utilization of inpatient service became less inequitable in 2008, the gap between the concentration indices of inpatient and outpatient services is still distinguished.

Recent attempts in China to increase coverage of insurance and primary healthcare could be a contributing factor to counteract the inequity of outpatient utilization, but better benefit packages and delivery strategies still need to be tested and scaled up to reduce future inequity in inpatient utilization in rural China.

## Competing interests

The authors declare that they have no competing interests.

## Authors’ contributions

Zhongliang Z, YS, JG and Zhengwei Z designed this study. Zhongliang Z, BC and YS reviewed literature. LX and YZ acquired data and provided administrative support for data analysis. Zhongliang Z analyzed the data and YS provided statistical expertise in reanalyzing the data. Zhongliang Z and YS interpreted the statistical results. Zhongliang Z, YS and BC drafted the manuscript. All authors critically revised the manuscript and approved the final manuscript. Zhongliang Z and YS made primary intellectual and practical contribution to this study and other co-authors made significant contribution as well.
